# *Tinospora cordifolia* attenuates cognitive impairment in rats with vascular dementia by suppressing TLR4/MyD88/NF‐κB signaling pathway induced neuroinflammation.

**DOI:** 10.1002/alz70858_102042

**Published:** 2025-12-25

**Authors:** Devesh Kumar, Gajendra T A, Sairam Krishnamurthy, Abhishek Pathak

**Affiliations:** ^1^ Institute of Medical Sciences, Banaras Hindu University, Varanasi, Uttar Pradesh, India; ^2^ Indian Institute of Technology (BHU), Varanasi, Uttar Pradesh, India

## Abstract

**Background:**

Vascular dementia (VaD) is an acquired syndrome and the second leading cause of dementia, following Alzheimer's disease (AD). It is characterized by neuroinflammation‐driven neurodegeneration, cognitive decline, and memory difficulties, particularly in the elderly. Unlike AD, no Food and Drug Administration‐approved treatments are available for VaD. *Tinospora cordifolia* (Guduchi or Gurjo), a medicinal herb renowned for its anti‐inflammatory and neuroprotective effects, has shown therapeutic potential in AD, but its application in VaD remains underexplored

**Method:**

The phytochemical composition of *T. cordifolia* ethanolic extract (TCEE) was analyzed using high‐resolution mass spectrometry after Soxhlet extraction. To induce the experimental model of VaD, rats underwent bilateral common carotid artery occlusion (BCCAO) and were subsequently divided into six groups. These groups were treated for 21 days with different interventions: sham‐operated, BCCAO, BCCAO + TCEE (at three varying doses), and BCCAO + Donepezil. To assess the effects of *T. cordifolia*, oxygen saturation was measured using ultrasound and photoacoustic imaging. Cognitive functions were evaluated using the Morris Water Maze, Open Field Test, and Novel Object Recognition. Neuronal morphological changes were examined through Hematoxylin‐Eosin (HE) and Nissl staining, while infarct size was quantified by 2,3,5‐triphenyl tetrazolium chloride (TTC) staining. Furthermore, inflammatory biomarkers were measured using enzyme‐linked immunosorbent assays.

**Result:**

In the VaD rat model, treatment with TCEE significantly enhanced cerebral blood flow, as shown by photoacoustic imaging, and reduced neuronal loss, as evidenced by Nissl, HE, and TTC staining. Cognitive performance also improved across various behavioral tests. *T. cordifolia* treatment mitigated glial activation, downregulated TLR4 and MyD88 expression, inhibited NF‐κB p65 phosphorylation, and reduced levels of pro‐inflammatory cytokines, including IL‐1β, IL‐6, and TNF‐α. Furthermore, similar effects were observed with the TLR4 inhibitor TAK‐242 and the acetylcholinesterase inhibitor Donepezil.

**Conclusion:**

The findings suggest that *T. cordifolia* may alleviate cognitive dysfunction in VaD by modulating the TLR4/MyD88/NF‐κB inflammatory signaling pathway. This herb shows promise as a potential therapeutic agent and TLR4 inhibitor for the treatment of VaD.

